# MiR-425 expression profiling in acute myeloid leukemia might guide the treatment choice between allogeneic transplantation and chemotherapy

**DOI:** 10.1186/s12967-018-1647-8

**Published:** 2018-10-01

**Authors:** Chen Yang, Tingting Shao, Huihui Zhang, Ninghan Zhang, Xiaoying Shi, Xuejiao Liu, Yao Yao, Linyan Xu, Shengyun Zhu, Jiang Cao, Hai Cheng, Zhiling Yan, Zhenyu Li, Mingshan Niu, Kailin Xu

**Affiliations:** 1 Blood Diseases Institute, Xuzhou Medical University, Xuzhou, Jiangsu China; 2 Department of Hematology, Affiliated Hospital of Xuzhou Medical University, Xuzhou, Jiangsu China; 3 Insititute of Nervous System Diseases, Xuzhou Medical University, Xuzhou, Jiangsu China; 4 School of Physical Science and Technology, ShanghaiTech University, Shanghai, China; 5 School of Life Science & Medicine, Dalian University of Technology, Panjin, China

**Keywords:** miR-425, Acute myeloid leukemia, Prognosis, Chemotherapy, Allo-HSCT

## Abstract

**Background:**

Acute myeloid leukemia (AML) is a highly heterogeneous disease. MicroRNAs function as important biomarkers in the clinical prognosis of AML.

**Methods:**

This study identified miR-425 as a prognostic factor in AML by screening the TCGA dataset. A total of 162 patients with AML were enrolled for the study and divided into chemotherapy and allogeneic hematopoietic stem cell transplantation (allo-HSCT) groups.

**Results:**

In the chemotherapy group, patients with high miR-425 expression had significantly longer overall survival (OS) and event-free survival (EFS) compared with patients with low miR-425 expression. In multivariate analyses, high miR-425 expression remained independently predictive of a better OS (HR = 0.502, *P* = 0.005) and EFS (HR = 0.432, *P* = 0.001) compared with patients with low miR-425 expression. Then, all patients were divided into two groups based on the median expression levels of miR-425. Notably, the patients undergoing allo-HSCT had significantly better OS (HR = 0.302, *P* < 0.0001) and EFS (HR = 0.379, *P* < 0.0001) compared with patients treated with chemotherapy in the low-miR-425-expression group. Mechanistically, high miR-425 expression levels were associated with a profile significantly involved in regulating cellular metabolism. Among these genes, MAP3K5, SMAD2, and SMAD5 were predicted targets of miR-425.

**Conclusions:**

The expression of miR-425 may be useful in identifying patients in need of strategies to select the optimal therapy between chemotherapy and allo-HSCT treatment regimens. Patients with low miR-425 expression may consider early allo-HSCT.

## Background

Acute myeloid leukemia (AML) originates from proliferative, clonal, and occasionally poorly differentiated cells of the hematopoietic system [1]. AML is the most common malignant myeloid disease in adults, accounting for 80% of adult leukemia [2]. Patients with AML showed heterogeneous outcomes after receiving different treatments, partly depending on patient age, karyotype, and mutational status. Conventional cytotoxic chemotherapy is the first-line treatment option for patients with AML. Allogeneic hematopoietic stem cell transplantation (allo-HSCT) offers strong anti-leukemic effect and potentially curative treatment in high-risk AML [3]. The prognosis of AML largely depends on treatment response and cytogenetic characteristics. Thus, the clinical and genetic prognostic markers are crucial in evaluating patients with AML and in guiding rational management.

Cytogenetics and molecular genetics were used for stratifying patients with AML into favorable, intermediate, and adverse prognostic risk groups [4]. Prognostic stratification in cytogenetically normal AML was mainly based on the mutational status of NPM1, FLT3, and CEBPA [5]. Patients with FLT3-ITD, RUNX1, ASXL1, and TP53 mutations were classified into the adverse risk group [5]. The decision to perform allo-HSCT depended on the assessment of the risk–benefit ratio based on cytogenetic and molecular genetic features. Patients with high-risk genetics were recommended to undergo allo-HSCT. However, patients with FLT3-ITD, TP53, and WT1 mutations still had poor outcome and high relapse rate even after allo-HSCT as a post-remission therapy. Currently, prognosis and optimal post-remission therapy cannot be predicted precisely in many subgroups of patients with AML [6, 7]. Therefore, novel prognostic markers are urgently needed to identify which patients are best suited for chemotherapy and who should be offered allo-HSCT.

MicroRNAs play important roles in modulating cellular behaviors by binding to the 3′-untranslated regions of their target mRNAs. MicroRNAs regulate the expression of intracellular proteins. They act as epigenetic regulators and influence the self-renewal and differentiation of leukemia stem cells [8]. Furthermore, the dysregulation of microRNA expression has been shown to be associated with the clinical outcome of patients with AML. MiR-181a has been confirmed to be associated with the favorable clinical outcome of patients with cytogenetically normal AML [9]. High miR-212 expression is independently predictive of favorable survival in AML [10]. In contrast, high miR-3151 expression was identified as an unfavorable prognosticator for patients with AML [11]. However, the relevance of microRNAs as predictive molecular markers to guide the treatment choice between allo-HSCT and chemotherapy is largely unknown. Therefore, the predictive microRNAs for the early and accurate identification of optimal therapy in patients with AML may help improve the clinical individual therapy.

In this study, miR-425 was identified as a prognostic factor in AML independent of other strong molecular predictors using genome-wide screening. A total of 162 patients newly diagnosed with AML were divided into two groups based on the allo-HSCT and chemotherapy treatment types. The prognostic role of miR-425 was analyzed in allo-HSCT and chemotherapy groups. A gene expression signature associated with miR-425 expression was derived in patients with AML to investigate biologic insights.

## Methods

### Patients

A total of 162 patients newly diagnosed with AML were recruited in this study. Seventy-two patients accepted allo-HSCT, and the remaining only accepted chemotherapy (with 3 days of an anthracycline and 7 days of cytarabine). All data sets are publicly available in the TCGA database. According to the Declaration of Helsinki, all participants included in this study signed the written informed consent, and the study was approved by the human studies committee of Washington University. The whole-genome and whole-exome sequencing analyses were performed to detect the mutational spectrum of patients with AML. The mRNA and miRNA sequencing were performed to analyze the expression of mRNAs and miRNAs. All clinical, molecular and cytogenetic information are publicly accessible from the TCGA website.

### Gene expression profiling

Of the 162 patients, 155 had both microRNA and mRNA expression data. These patient samples were used for identifying the gene expression profile associated with miR-425 expression. For microRNA-seq data, read counts for each sample were normalized to reads per million. For expression profiling, the expression values were logged (base 2) before analysis. A comparison of mRNA expression was made between patients with high and low microRNA expression. Finally, gene rows were reordered using a hierarchical clustering analysis. The online applications miRBase Targets Version 7 and Targetscan Release 7.1 were used for the in silico target prediction of microRNAs. The gene/microRNA expression signatures were derived by Spearman correlation analysis. Gene ontology enrichment analysis of genes in miR-425-associated signature was conducted using the Database for Annotation, Visualization, and Integrated Discovery.

### Definition of clinical endpoints and statistical analysis

Overall survival (OS) was measured from the date of diagnosis to the date of death at the last follow-up. Event-free survival (EFS) was measured from the date of diagnosis to the date when the first adverse event, including relapse and death, occurred. Pearson Chi square and Fisher’s exact tests were used for categorical variables. The Mann–Whitney *U* test was performed for analyzing continuous variables. Estimated distributions of OS and EFS were calculated using the Kaplan–Meier method, and the log-rank test was used to compare differences between survival curves. In univariate and multivariate analyses, Cox proportional hazards models were used to test the relationship between survival and miR-425. The hazard ratio (HR) and its 95% confidence interval (CI) were assessed using the Cox proportional hazards model. All statistical tests were performed as two sided, and a *P* value < 0.05 was considered statistically significant. SPSS software 18.0 and GraphPad Prism software 6.0 were used for the statistical analysis in this study.

## Results

### Associations of miR-425 expression with clinical and molecular features

Details on the molecular and clinical characteristics of the patients are summarized in Table 1. Pearson Chi square test, Fisher’s exact test, and Mann–Whitney *U* test were performed to investigate the associations of miR-425 with clinical and molecular characteristics. The median expression level of miR-425 was used to define high- and low-miR-425-expression groups. In the chemotherapy group, patients with low miR-425 expression were more often diagnosed with M0 compared with patients with high miR-425 expression (*P* = 0.006). Moreover, patients with higher miR-425 expression in the chemotherapy group had a lower BM (*P* = 0.050) and PB blast (*P* = 0.002) counts. Patients with miR-425 upregulation included more good risk cases (*P* = 0.002). AML is a complex disease, characterized by multiple somatically acquired driver mutations. Genetic abnormalities are powerful prognostic factors for AML. Thus, we analyzed if miR-425 expression associated with the gene mutation status. Patients with higher miR-425 expression often had more CBFβ-MYH11 (*P* = 0.012) mutation and fewer IDH1 (*P* = 0.012) mutation. No significant differences were observed in mutation frequencies of NPM1, DNMT3A, RUNX1, MLL-PTD, IDH1, IDH2, and TP53 between the two groups (Table 1). These data suggest that the prognostic role of miR-425 expression may be independently associated with these gene mutations. In the allo-HSCT group, no significant differences were found in terms of age, gender, white blood cell count, BM blast, PB blast, NPM1, DNMT3A, RUNX1, MLL-PTD, IDH1, IDH2, and TP53 between high- and low-miR-425 expression groups.

**Table 1:** Comparison of clinical and molecular characteristics with miR-425 expression in AML patients

Characteristic	Chemotherapy group			Allo-HSCT group		
	High miR-425 (n = 45)	Low miR-425 (n = 45)	*P*	High miR-425 (n = 36)	Low miR-425 (n = 36)	*P*
Age/years, median (range)	63 (22–88)	68 (33–83)	0.138	52 (25–72)	50 (18–69)	0.450
Age group/no. (%), years			0.652			0.793
< 60	16 (35.6)	13 (28.9)		27 (75.0)	25 (69.4)	
≥ 60	29 (64.4)	32 (71.1)		9 (25.0)	11 (30.6)	
Gender/no. (%)			0.289			1.000
Male	28 (62.2)	22 (48.9)		21 (58.3)	20 (55.6)	
Female	17 (37.8)	23 (51.1)		15 (41.7)	16 (44.4)	
WBC/× 10^9^/L, median (range)	15.2 (0.7–298.4)	16.9 (1.0–297.4)	0.589	30.3 (1.5–98.8)	28.3 (0.6–223.8)	0.978
BM blast/ %, median (range)	67 (30–98)	75 (37–99)	0.050	68 (30–97)	76 (35–100)	0.131
PB blast/%, median (range)	18 (0–71)	53 (0–98)	0.002	41 (0–85)	53 (0–96)	0.116
FAB subtypes/no. (%)						
M0	0 (0.0)	8 (17.8)	0.006	3 (8.3)	6 (16.7)	0.478
M1	7 (15.6)	13 (28.9)	0.201	8 (22.2)	15 (41.7)	0.129
M2	13 (28.9)	8 (17.8)	0.231	13 (36.1)	6 (16.7)	0.107
M4	14 (31.1)	10 (22.2)	0.340	8 (22.2)	6 (16.7)	0.767
M5	9 (20)	4 (8.9)	0.230	3 (8.3)	1 (2.8)	0.614
M6	0 (0.0)	1 (2.2)	1.000	1 (2.8)	0 (0.0)	1.000
M7	1 (2.2)	1 (2.2)	1.000	0 (0.0)	1 (2.8)	1.000
Others	1 (2.2)	0 (0.0)	1.000	0 (0.0)	1 (2.8)	1.000
Karyotype/no. (%)						
Normal	21 (46.7)	23 (51.1)	0.833	17 (47.2)	17 (47.2)	1.000
Complex	5 (11.1)	7 (15.6)	0.758	5 (13.9)	7 (19.4)	0.753
MLL rearranged	2 (4.4)	1 (2.2)	1.000	2 (5.6)	1 (2.8)	1.000
CBFβ-MYH11	7 (15.6)	0 (0.0)	0.012	5 (13.9)	0 (0.0)	0.054
BCR-ABL1	1 (2.2)	0 (0.0)	1.000	1 (2.8)	1 (2.8)	1.000
RUNX1-RUNX1T	5 (11.1)	1 (2.2)	0.203	0 (0.0)	1 (2.8)	1.000
Others	4 (8.9)	13 (28.9)	0.029	6 (16.7)	9 (25)	0.563
Risk (cyto)/no. (%)						
Good	12 (26.7)	1 (2.2)	0.002	5 (13.9)	1 (2.8)	0.199
Intermediate	20 (44.4)	30 (66.7)	0.056	17 (47.2)	24 (66.7)	0.153
Poor	13 (28.9)	12 (26.7)	1.000	13 (36.1)	11 (30.6)	0.803
Others	0 (0.0)	2 (4.4)	0.494	1 (2.8)	0 (0.0)	1.000
FLT3-ITD/no. (%)			1.000			0.396
Presence	8 (17.8)	8 (17.8)		6 (16.7)	10 (27.8)	
Absence	37 (82.2)	37 (82.2)		30 (83.3)	26 (72.2)	
NPM1/no. (%)			0.367			0.430
Presence	12 (26.7)	17 (37.8)		8 (22.2)	12 (33.3)	
Absence	33 (73.3)	28 (62.2)		28 (77.8)	24 (66.7)	
DNMT3A/no. (%)			0.059			0.415
Presence	8 (17.8)	17 (37.8)		7 (19.4)	11 (30.6)	
Absence	37 (82.2)	28 (62.2)		29 (80.6)	25 (69.4)	
RUNX1/no. (%)			0.058			1.000
Presence	1 (2.2)	7 (15.6)		4 (11.1)	4 (11.1)	
Absence	44 (97.8)	38 (84.4)		32 (88.9)	32 (88.9)	
MLL-PTD/no. (%)			1.000			0.115
Presence	2 (4.4)	3 (6.7)		4 (11.1)	0 (0.0)	
Absence	43 (95.6)	42 (93.3)		32 (88.9)	36 (100.0)	
TP53/no. (%)			1.000			1.000
Mutation	5 (11.1)	5 (11.1)		2 (5.6)	2 (5.6)	
Wild type	40 (88.9)	40 (88.9)		34 (94.4)	34 (94.4)	
CEBPA/no. (%)			1.000			1.000
Mutation	2 (4.4)	1 (2.2)		4 (11.1)	4 (11.1)	
Wild type	43 (95.6)	44 (97.8)		32 (89.9)	32 (88.9)	
IDH1/no. (%)			0.012012			0.514
Mutation	0 (0.0)	7 (15.6)		4 (11.1)	7 (19.4)	
Wild type	45 (100.0)	38 (84.4)		32 (88.9)	29 (80.6)	
IDH2/no. (%)			1.000			0.710
Mutation	4 (8.9)	5 (11.1)		3 (8.3)	5 (13.9)	
Wild type	41 (91.1)	40 (88.9)		33 (91.7)	31 (86.1)	

*WBC* white blood cell, *BM* bone marrow, *PB* peripheral blood, *FAB* French–American–British classification, *MLL* mixed-lineage leukemia, *FLT3-ITD* internal tandem duplication of the FLT3 gene, *NPM1* nucleophosmin, *DNMT3A* DNA methyltransferase 3A, *RUNX1* runt related transcription factor 1, *MLL-PTD* partial tandem duplication of the MLL gene, *CEBPA* CCAAT/enhancerbinding protein α, *IDH* isocitrate dehydrogenase

The median expression level of miR-425 was used to define high- and low-miR-425-expression groups. *P* values for continuous variables are from Mann–Whitney test; *P* values for categorical variables are from Chi square tests. The values represent frequencies (%). Complex karyotype is defined as more than or equal to 3 chromosomal abnormalities. The patients were divided into three risk groups (good, intermediate and poor) according to the cytogenetic risk classification

### Prognostic value of miR-425 in patients with chemotherapy and allo-HSCT treatment

**Fig. 1:**
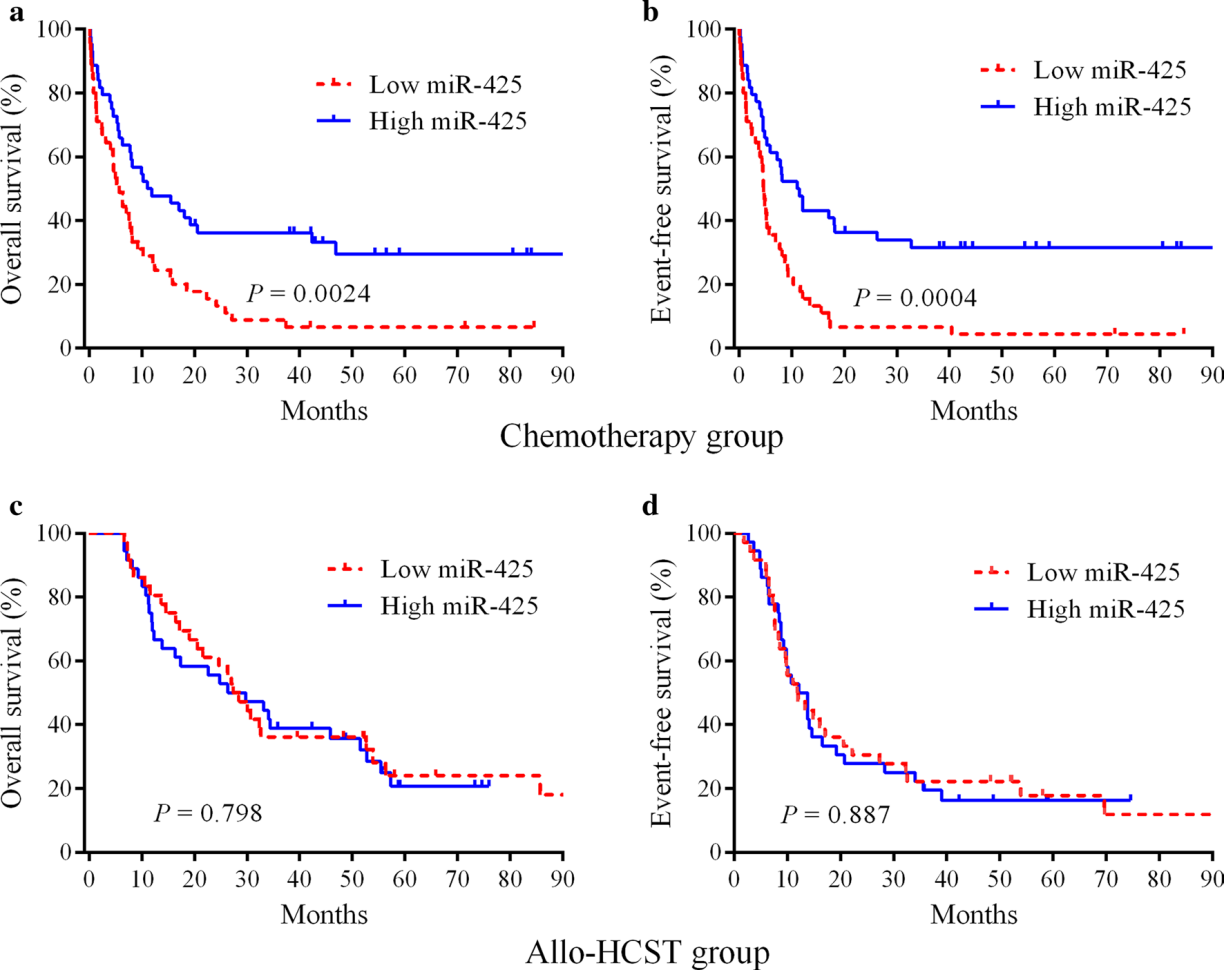
Kaplan–Meier survival curves of patients with respect to miR-425 expression. **a**, **b** Patients with high miR-425 expression had significantly longer OS and EFS in the chemotherapy group (*n* = 90). **c**, **d** Effect of miR-425 expression on OS and EFS in patients undergoing allo-HSCT (*n* = 72)

Kaplan–Meier curves and log-rank test were used to test for differences in survival distribution so as to investigate the prognostic role of miR-425 in patients with AML. In the chemotherapy group, the survival distribution curves showed that patients with high miR-425 expression had significantly longer OS (*P* = 0.0024) and EFS (*P* = 0.0004) compared with patients with low miR-425 expression (Fig. 1a, b). However, no significant differences were found between high- and low-miR-425-expression groups in patients undergoing allo-HSCT (Fig. 1c, d). These data suggested that high expression of miR-425 specifically predicted a favorable outcome in patients with AML treated with chemotherapy.

### MiR-425 was independently associated with a clinical outcome in AML

Univariate and multivariate analyses were performed to assess the value of miR-425 in predicting the OS and EFS of patients with AML. In the chemotherapy group, the univariate analysis showed that the high expression of miR-425 had a prognostic value for predicting EFS (HR = 0.449, 95% CI = 0.279–0.721, *P* = 0.001) and OS (HR = 0.506, 95% CI = 0.316–0.811, *P* = 0.005). In the multivariate analysis, miR-425 and several well-known prognostic factors were included into the model (Table 2). High miR-425 remained independently predictive of longer EFS (HR = 0.432, 95% CI = 0.266–0.703, *P* = 0.001) after adjusting for TP53 (*P* = 0.004) and CEBPA (*P* = 0.034). High miR-425 expression was also independently predictive of longer OS (HR = 0.502, 95% CI = 0.311–0.811, *P* = 0.005) after adjusting for the P53 mutational status (HR = 2.519, 95% CI = 1.286–4.933, *P* = 0.007).

**Table 2:** Univariate and multivariate analyses in patients treated with chemotherapy

Variables	EFS		OS	
	HR (95% CI)	*P*-value	HR (95% CI)	*P*-value
Univariate analyses				
MiR-425 (high vs. low)	0.449 (0.279–0.721)	0.001	0.506 (0.316–0.811)	0.005
WBC (≥ 20 vs. < 20 × 10^9^/L)	0.939 (0.594–1.484)	0.786	0.936 (0.591–1.484)	0.779
FLT3-ITD (positive vs. negative)	1.242 (0.693–2.224)	0.467	1.192 (0.665–2.136)	0.555
NPM1 (mutated vs. wild)	1.168 (0.721–1.893)	0.527	1.044 (0.640–1.704)	0.862
DNMT3A (mutated vs. wild)	1.491 (0.909–2.446)	0.114	1.432 (0.868–2.362)	0.160
RUNX1 (mutated vs. wild)	1.464 (0.700–3.064)	0.312	1.591 (0.759–3.335)	0.219
TP53 (mutated vs. wild)	2.949 (1.510–7.561)	0.002	2.898 (1.487–5.694)	0.002
CEBPA (mutated vs. wild)	2.963 (0.910–9.648)	0.071	2.901 (0.892–9.434)	0.077
IDHI/IDH2 (mutated vs. wild)	1.006 (0.570–1.775)	0.983	0.954 (0.532–1.710)	0.874
NRAS/KRAS (mutated vs. wild)	1.143 (0.601–2.172)	0.684	1.188 (0.625–2.260)	0.599
MLL-PTD (mutated vs. wild)	0.836 (0.305–2.291)	0.727	0.910 (0.332–2.495)	0.855
Multivariate analyses				
MiR-425 (high vs. low)	0.432 (0.266–0.703)	0.001	0.502 (0.311–0.811)	0.005
FLT3-ITD (positive vs. negative)	1.582 (0.868–2.883)	0.134	–	–
TP53 (mutated vs. wild)	2.737 (1.377–5.438)	0.004	2.519 (1.286–4.933)	0.007
CEBPA (mutated vs. wild)	3.746 (1.102–12.736)	0.034	3.228 (0.959–10.864)	0.058

*EFS* event-free survival, *OS* overall survival, *HR* hazard ratio, *CI* confidence interval, *WBC* white blood cell, *FLT3-ITD* internal tandem duplication of the FLT3 gene, *NPM1* nucleophosmin, *DNMT3A* DNA methyltransferase 3A, *RUNX1* runt related transcription factor 1, *MLL-PTD* partial tandem duplication of the MLL gene, *CEBPA* CCAAT/enhancerbinding protein α, *IDH* isocitrate dehydrogenase

Cox proportional hazards model was used for EFS and OS. HRs greater than 1.0 indicate higher and those less than 1.0 indicate lower risk for EFS or OS

In the allo-HSCT group, the univariate analysis showed that patients with MLL-PTD had shorter EFS (HR = 6.028, *P* = 0.001) and OS (HR = 3.106, *P* = 0.032). FLT3-ITD was unfavorable for both EFS (HR = 1.873, *P* = 0.043) and OS (HR = 1.998, *P* = 0.034). Mutations in TP53 (HR = 6.028, *P* = 0.001) and RUNX1 (HR = 6.028, *P* = 0.001) were unfavorable for OS (Table 3). The multivariate analysis indicated that FLT3-ITD (HR = 2.549, *P* = 0.006), TP53 (HR = 5.841, *P* = 0.002), and RUNX1 (HR = 3.007, *P* = 0.007) mutational status remained to be an independent prognostic factor for OS (Table 3). However, no notable differences in survival were reported between high- and low-miR-425-expression groups.

**Table 3:** Univariate and multivariate analyses in patients treated with allo-HSCT

Variables	EFS		OS	
	HR (95% CI)	*P*-value	HR (95% CI)	*P*-value
Univariate analyses				
MiR-425 (high vs. low)	1.037 (0.624–1.724)	0.888	1.073 (0.626–1.840)	0.798
WBC (≥ 20 vs. < 20 × 10^9^/L)	1.530 (0.910–2.571)	0.108	0.949 (0.554–1.628)	0.851
FLT3-ITD (positive vs. negative)	1.873 (1.020–3.437)	0.043	1.998 (1.053–3.788)	0.034
NPM1 (mutated vs. wild)	0.913 (0.515–1.619)	0.755	0.879 (0.478–1.671)	0.678
DNMT3A (mutated vs. wild)	1.106 (0.615–1.989)	0.737	1.269 (0.686–2.347)	0.447
RUNX1 (mutated vs. wild)	1.375 (0.650–2.907)	0.404	2.253 (1.046–4.849)	0.038
TP53 (mutated vs. wild)	1.579 (0.565–4.411)	0.383	3.788 (1.289–11.133)	0.015
CEBPA (mutated vs. wild)	0.853 (0.366–1.989)	0.713	0.644 (0.256–1.620)	0.350
IDHI/IDH2 (mutated vs. wild)	0.761 (0.417–1.389)	0.374	0.802 (0.422–1.524)	0.500
NRAS/KRA S (mutated vs. wild)	1.373 (0.622–3.034)	0.433	0.658 (0.261–1.657)	0.374
MLL-PTD (mutated vs. wild)	6.093 (2.051–18.098)	0.001	3.106 (1.104–8.741)	0.032
Multivariate analyses				
WBC (≥ 20 vs. < 20 × 10^9^/L)	1.691 (0.973–2.940)	0.062	–	–
FLT3-ITD (positive vs. negative)	1.740 (0.934–3.239)	0.081	2.549 (1.306–4.975)	0.006
TP53 (mutated vs. wild)	2.657 (0.877–8.048)	0.084	5.841 (1.895–18.009)	0.002
RUNX1 (mutated vs. wild)	–	–	3.007 (1.355–6.673)	0.007
MLL-PTD (mutated vs. wild)	6.028 (2.001–18.158)	0.001	–	–

*EFS* event-free survival, *OS* overall survival, *HR* hazard ratio, *CI* confidence interval, *WBC* white blood cell, *FLT3-ITD* internal tandem duplication of the FLT3 gene, *NPM1* nucleophosmin, *DNMT3A* DNA methyltransferase 3A, *RUNX1* runt related transcription factor 1, *MLL-PTD* partial tandem duplication of the MLL gene, *CEBPA* CCAAT/enhancerbinding protein α, *IDH* isocitrate dehydrogenase

Cox proportional hazards model was used for EFS and OS. HRs greater than 1.0 indicate higher and those less than 1.0 indicate lower risk for EFS or OS

### Patients with low expression of miR-425 benefited from allo-HSCT treatment

**Fig. 2:**
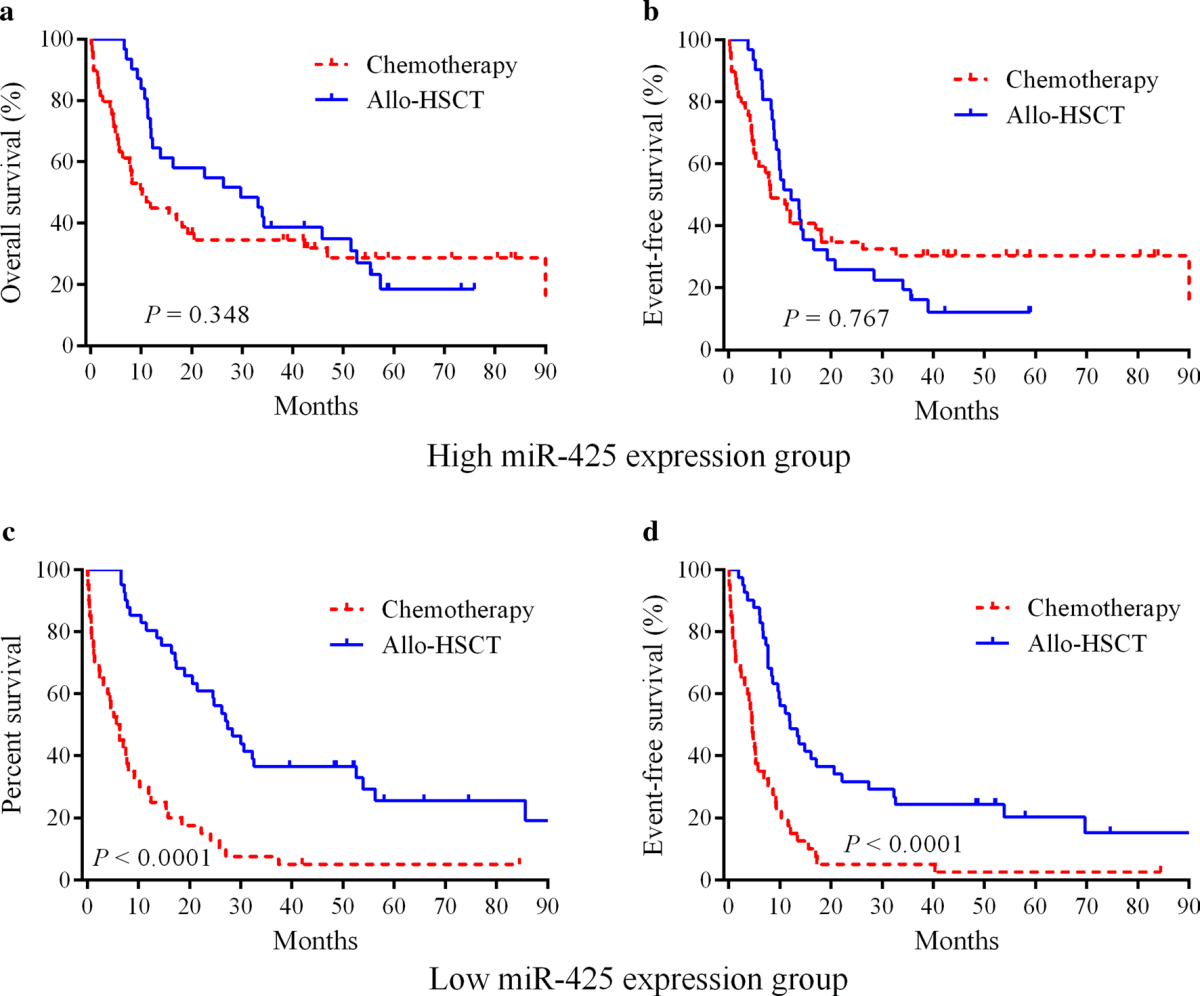
Allo-HSCT overcame the adverse prognosis of low miR-425 expression in AML.**a**, **b** A total of 162 patients were divided into two groups based on the median expression levels of miR-425. Kaplan–Meier survival curves of patients with respect to chemotherapy (*n* = 40) and allo-HSCT (*n* = 41) treatment in the low-miR-425-expression group. **c**, **d** Kaplan–Meier survival curves of patients with respect to chemotherapy (*n* = 50) and allo-HSCT (*n* = 31) treatment in the high-miR-425-expression group

All 162 patients were divided into two groups based on the median expression levels of miR-425 to investigate whether allo-HSCT could overcome the adverse outcome of low miR-425 expression. In the low-miR-425-expression group, the patients undergoing allo-HSCT had significantly better OS (HR = 0.302, 95% CI = 0.129–0.375, *P* < 0.0001) and EFS (HR = 0.379, 95% CI = 0.192–0.523, *P* < 0.0001) compared with patients treated with chemotherapy (Fig. 2c, d). In the high-miR-363-expression group, no significant differences in OS (*P* = 0.127) and EFS (*P* = 0.226) were observed between chemotherapy and allo-HSCT treatment types (Fig. 2a, b).

### Biologic Insights

**Fig. 3:**
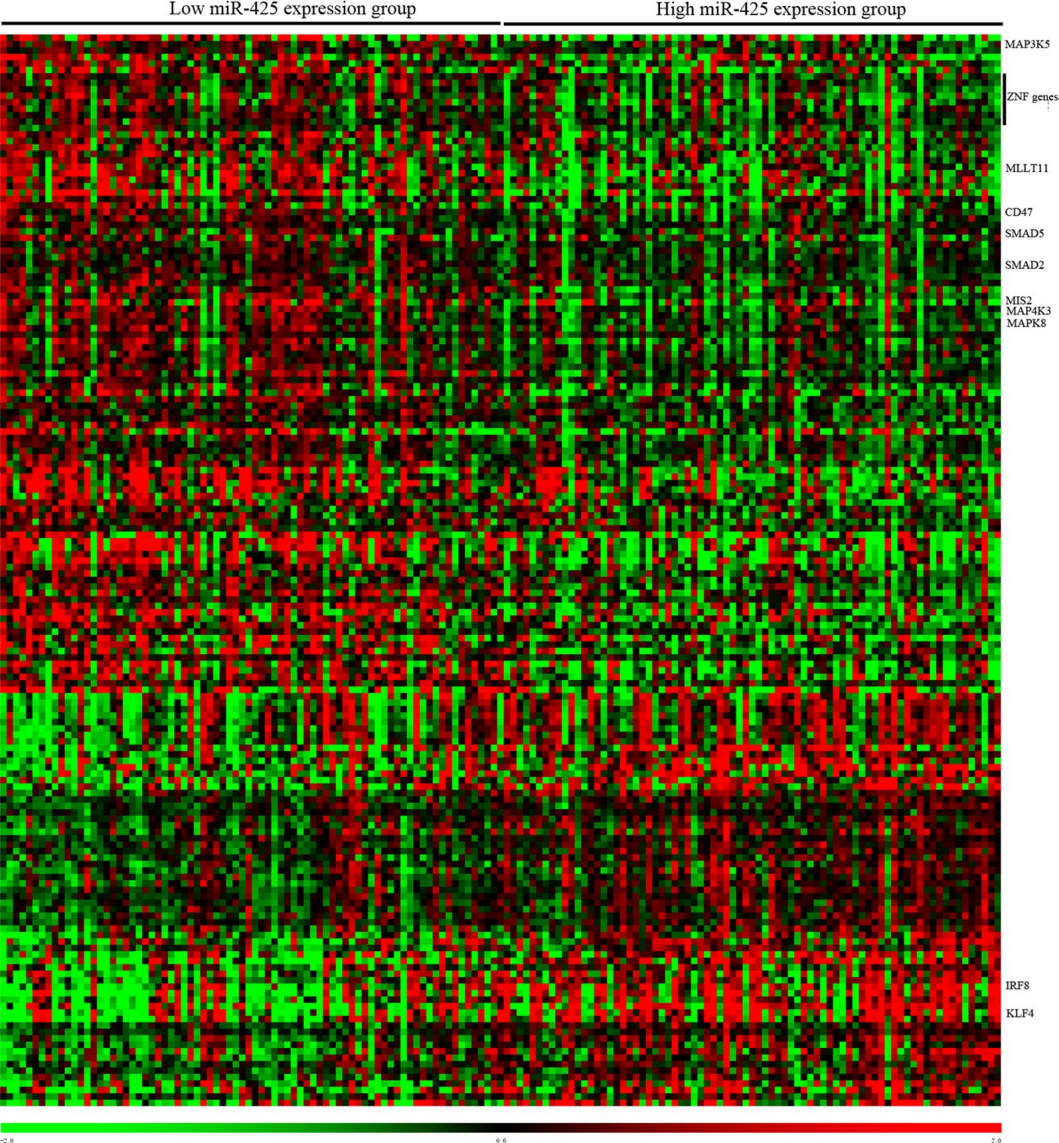
Heat map of gene expression profile associated with miR-425 expression in patients with AML. Patients (columns) are ordered from left to right by increasing miR-425 expression levels. Genes (rows) are ordered by the hierarchical cluster analysis. Green color indicates expression levels lower than the median value for the given gene set, and red color indicates expression levels higher than the median value for the given gene set. The miR-425-correlated genes mentioned in the text are indicated

To gain insights into the biological function of miR-425, gene/microRNA expression signatures were derived using Spearman correlation analysis. We observed that the mRNA expression of 166 genes significantly correlated (*P* < 0.001) with that of miR-425. Of these genes, 102 correlated negatively and 64 correlated positively (Fig. 3). MiR-425 expression negatively correlated with the levels of the CD47, SMAD2, SMAD5, MAP4K3, MAP3K5, MAPK8, MLLT11, MIS2, and zinc finger protein family genes. Interestingly, MAP3K5, SMAD2, and SMAD5 were in silico predicted targets of miR-425. Also, a negative correlation of miR-425 with the expression of IRF8 and KLF4 was found. Furthermore, the Gene Ontology analysis revealed that the genes involved in biologic processes, including cellular metabolism, macromolecule metabolism, RNA metabolism, cellular biosynthesis, embryo development, cell division, and protein phosphorylation, were significantly over-represented among the differentially expressed genes correlated with miR-425 expression (Table 4).

**Table 4:** Gene ontology terms of biological processes in the miR-425 associated expression profile

GO ID	GO terms	Percentage of Members of the GO Term Present in the miR-425 Profile	*P*-value
GO:0008152	Metabolic process	62.8	0.004
GO:0044237	Cellular metabolic process	59.6	0.002
GO:0043170	Macromolecule metabolic process	55.7	< 0.001
GO:0044267	Cellular protein metabolic process	30.7	0.029
GO:0031326	Regulation of cellular biosynthetic process	29.4	0.006
GO:0009889	Regulation of biosynthetic process	29.4	0.008
GO:0010468	Regulation of gene expression	29.4	0.009
GO:0051252	Regulation of RNA metabolic process	25.0	0.026
GO:0065009	Regulation of molecular function	20.5	0.019
GO:0006468	Protein phosphorylation	15.3	0.011
GO:0009790	Embryo development	8.9	0.026
GO:0051301	Cell division	7.7	0.004
GO:0032101	Regulation of response to external stimulus	7.7	0.019
GO:0010608	Posttranscriptional regulation of gene expression	5.8	0.025
GO:0042787	Protein ubiquitination	3.8	0.026

*GO* gene ontology

## Discussion

Identification of novel predictive markers to guide clinical therapy for patients with AML is currently of special interest. This study reported that miR-425 served as an independent prognostic factor in patients with AML. Furthermore, the results suggested that low miR-425 expression correlated with an adverse outcome in patients with AML treated with chemotherapy. However, allo-HSCT could remarkably overcome the adverse effect of low miR-425 expression. Thus, miR-425 might be considered as a predictive molecular marker to guide the treatment choice between allo-HSCT and chemotherapy.

Effective prognostic markers to guide the selection of appropriate therapy for patients with AML are lacking. In the present study, the high miR-425 expression could predict the favorable outcome when other molecular prognostic markers were considered in multivariable models. Thus, miR-425 might add to the prognostic effect of different previously established molecular markers in a highly mixed population of AML. These results provided an opportunity for potential therapeutic intervention with synthetic miR-425 compounds. More importantly, this study found that patients with low miR-425 expression undergoing allo-HSCT had significantly better OS and EFS compared with patients treated with chemotherapy. Among the patients with high miR-425 expression, no benefit was reported for the allo-HSCT group compared with the chemotherapy group. These finding suggested that patients with high miR-425 expression might not benefit from allo-HSCT as the first-line treatment. Thus, the expression of miR-425 might be useful for identifying patients in need of strategies to choose the better treatment types between chemotherapy and allo-HSCT. Patients with low miR-425 expression may warrant the strong consideration of early allo-HSCT.

Allo-HSCT has been confirmed to improve the prognosis of AML with gene mutations. TP53 is an inactivated tumor suppressor gene. The mutations of TP53 are often observed in advanced- or complex-karyotype AML. However, a previous study showed that even after allogeneic HSCT, patients harboring mutated p53 showed a dismal result [12]. The analysis results also suggested that mutations in TP53 were associated with a poor outcome even after allo-HSCT. The TP53 mutation was reported to induce drug resistance, eventually contributing to treatment failure [13]. TP53 is regulated by the PI3 K-Akt pathway. Therefore, blocking the activity of PI3 K-Akt pathway might increase p53 activity, eventually increasing chemosensitivity [14]. A recent study demonstrated that miR-425 further inhibited the PI3K-Akt pathway via targeting the IGF1 [15]. This function of miR-425 might contribute to the better response to chemotherapy in patients with AML having high miR-425 expression.

The activity of miR-425 has been recently characterized in solid tumors. Elevated miR-425 has been observed in gastric cancer, where it promotes cell proliferation and inhibits apoptosis by targeting PTEN [16]. A later study revealed that miR-425 was also involved in gastric cancer progression and metastasis by suppressing CYLD [17]. In breast cancer, miR-425 promoted cell proliferation through suppressing EGR1 [18]. Lower miR-425 limited the migration and invasion ability of esophageal squamous cell carcinoma [19]. MiR-425 has been found to be capable of inhibiting melanoma metastasis through repressing the PI3 K-Akt pathway by targeting IGF-1 in melanoma [15]. However, relatively little is known about the biological role of miR-425 in AML.

The genes significantly correlated with miR-425 expression were identified to further understand how miR-425 expression affected the response to treatment and clinical outcome of patients with AML. The Gene Ontology analysis revealed that the genes involved in metabolic processes significantly correlated with miR-425 expression. The dysregulated cellular metabolism is now widely accepted as an emerging hallmark of AML. The aberrant metabolism is crucial in leukemogenesis and chemoresistance with the power to control both genetic and epigenetic events in AML [20]. Furthermore, the miR-425 level was found to be negatively correlated with the expression of leukemogenic genes, including CD47, SMAD2, SMAD5, MAP4K3, MAP3K5, MAPK8, and MLLT11. Available evidence proves that CD47 help leukemia cells to avoid phagocytosis [21–23]. MAP4K3, as direct LATS1/2-activating kinase, contributes to core components of the Hippo pathway [24] and participates in amino acid signaling [25]. MAP3K5, as an apoptosis signal-related kinase, may be inactivated by Akt [26]. A previous study revealed the role of MAPK8 in controlling autophagy [27]. MLLT11, as an MLL fusion partner, has shown poor survival benefits in leukemias [28]. SMAD2, as a TGFƁ receptor–associated signaling molecule, is involved in TGFB-induced growth arrest [29]. It is also crucial in the chemoresistance of leukemic cells caused by hypoxia [30, 31]. High expression of SMAD5 has been found to be associated with the proliferation and differentiation of osteoblasts in regulating leukemia [32]. Notably, MAP3K5, SMAD2, and SMAD5 were predicted to be the targets of miR-425. Furthermore, a positive correlation of miR-425 expression with IRF8 and KLF4 was found. IRF8 showed an inverse correlation with WT1, which had a worse effect on recurrence-free survival and OS [33]. Increasing evidence links KLF4 to myeloid leukemias [34]. It exerts a powerful anti-leukemic effect by regulating microRNA and gene targets [35], suggesting that KLF4 functions as a tumor suppressor in leukemia. Taken together, the miR-425-associated gene expression profiling analyses provided insights into the leukemogenic role of genes that are either direct or indirect targets of miR-425. These miR-425-associated genes might support the clinical observation of AML defined by miR-425 expression.

Our analysis was based on information obtained from The Cancer Genome Atlas (TCGA) database. The strengths of the trial included its well-defined eligibility criteria and uniform treatment regimens in accordance with NCCN guidelines. Furthermore, the study was designed and powered to detect > 99% of mutations that are present in at least 5% of all de novo AML cases; cases were chosen from a collection of more than 400 consented AML samples to represent the currently recognized subtypes of the disease (based on morphologic and cytogenetic criteria). However, the data also presented some key limitations for analysis. Our analysis is a retrospective design. It is restricted to available data and their inherent limitations. The small sample size may reduce the accuracy of our results. Therefore, the present results need to be verified in larger cohorts.

## Conclusions

The expression of miR-425 was independently associated with the clinical outcome in a highly heterogeneous population of AML. The expression analysis of miR-425 might help improve the risk stratification and decision making regarding the treatment of patients with AML. Furthermore, allo-HSCT might overcome the adverse outcome associated with the low expression of miR-425 in AML. Thus, the expression of miR-425 might be useful for identifying patients in need of strategies to choose better therapies between chemotherapy and allo-HSCT.

## Abbreviations

AML: acute myeloid leukemia
allo-HSCT: allogeneic hematopoietic stem cell transplantation
EFS: event-free survival
OS: overall survival
WBC: white blood cell
BM: bone marrow
PB: peripheral blood
FAB: French–American–British classification
MLL: mixed-lineage leukemia
FLT3-ITD: internal tandem duplication of the FLT3 gene
NPM1: nucleophosmin
DNMT3A: DNA methyltransferase 3A
RUNX1: runt related transcription factor 1
MLL-PTD: partial tandem duplication of the MLL gene
CEBPA: CCAAT/enhancerbinding protein α
IDH: isocitrate dehydrogenase
